# Brain volume is related to neurological impairment and to copper overload in Wilson’s disease

**DOI:** 10.1007/s10072-019-03942-z

**Published:** 2019-05-30

**Authors:** Lukasz Smolinski, Tomasz Litwin, Barbara Redzia-Ogrodnik, Karolina Dziezyc, Iwona Kurkowska-Jastrzebska, Anna Czlonkowska

**Affiliations:** 1 Second Department of Neurology, Institute of Psychiatry and Neurology, Sobieskiego 9, 02-957 Warsaw, Poland; 2 Department of Radiology, Institute of Psychiatry and Neurology, Warsaw, Poland

**Keywords:** Wilson’s disease, Unified Wilson’s disease rating scale, Volumetric MRI

## Abstract

**Introduction:**

To determine whether brain volume was associated with functional and neurological impairments and with copper overload markers in patients with Wilson’s disease.

**Methods:**

In 48 treatment-naïve patients, we assessed functional and neurological impairments with the Unified Wilson’s Disease Rating Scale, measured normalized brain volumes based on magnetic resonance images, and assessed concentration of non-ceruloplasmin-bound copper. We correlated brain volume measures with functional and neurological impairment scores and copper overload indices.

**Results:**

Functional and neurological impairments correlated with all brain volume measures, including the total brain volume and the volumes of white matter and gray matter (both peripheral gray matter and deep brain nuclei). Higher non-ceruloplasmin-bound copper concentrations were associated with greater functional and neurological impairments and lower brain volumes.

**Conclusions:**

Our findings provided the first in vivo evidence that the severity of brain atrophy is a correlate of functional and neurological impairments in patients with Wilson’s disease and that brain volume could serve as a marker of neurodegeneration induced by copper.

**Electronic supplementary material:**

The online version of this article (10.1007/s10072-019-03942-z) contains supplementary material, which is available to authorized users.

## Introduction

Wilson’s disease (WD) is an autosomal recessive disorder caused by mutations in the gene that encodes ATPase 7B (ATP7B), the membrane-bound copper transporter [1]. This enzyme, expressed in the liver, plays a central role in copper trafficking. It delivers copper to apoceruloplasmin and excretes excess dietary copper into the bile [2]. In WD, dysfunction of ATP7B leads to hypoceruloplasminemia and insufficient copper removal from liver cells, which results in liver injury; consequently, copper is released into the blood in the form of non-ceruloplasmin-bound copper (NCC), which accumulates and causes damage to other tissues, particularly the brain [2]. Thus, patients with WD present with hepatic and/or neurological symptoms and may have corneal copper deposits (Kayser-Fleisher rings) [1, 2].

On brain magnetic resonance images (MRIs) of patients with WD, bilateral T2-hyperintensities are typically observed in the deep brain structures [3–5], and widespread brain atrophy is also commonly observed [5–7]. Compared to healthy controls, brains of patients with WD display reduced volumes in the caudate nuclei, globi pallidi, thalami, cerebellum, and cerebral cortex [8]. Previous research has shown that brain volume correlates with clinical impairment in different neurological diseases. In multiple sclerosis, whole brain volume, the volumes of gray and white matter, and thalamic volume correlate with disease severity [9]. In Alzheimer’s disease, hippocampal volume is related to cognitive function [10], and motor impairment is related to whole brain volume and the volume of gray matter in patients with progressive supranuclear palsy [11]. In this study, we aimed to determine whether a similar relationship is observed in WD. To that end, we correlated brain volume measures with functional and neurological impairments, assessed using the Unified Wilson’s Disease Rating Scale (UWDRS), [12, 13] in a relatively large group of treatment-naive patients with WD. We also investigated whether brain atrophy was associated with indices of copper overload.

## Methods

### Participants

In this cross-sectional study, we analyzed prospectively collected data of 48 patients with WD. We included consecutive treatment-naïve adult patients diagnosed with WD in our department, after the introduction of a consistent brain imaging protocol, between December 2011 and December 2016. WD was diagnosed and classified into one of three clinical phenotypes (i.e., presymptomatic, predominantly hepatic, and predominantly neurological) according to international recommendations [14]. In addition, patients with the neurological phenotype were further classified based on the predominant neurological syndrome type based on classifications described by Marsden [15] and Oder et al. [16], i.e., tremor (including patients with predominant tremor and ataxia), parkinsonism (including rigidity, rest tremor and hypokinesia), and dystonia (including choreoathetosis) [17]. Disease duration was defined as the time from first hepatic or neurological symptoms, which did not prompt a diagnostic work-up, to the time of diagnosis. The presence of corneal copper deposits in either eye (the Kayser-Fleischer ring) was determined by an ophthalmologist in a slit-lamp examination. The study was approved by the Bioethics Committee of the Institute of Psychiatry and Neurology, Warsaw. We included only data of patients that provided signed consent to use their clinical and laboratory data for research purposes.

### Rating of neurological and functional impairment

Before treatment initiation, the UWDRS [12, 13] was used to rate functional impairment (UWDRS_FI, items 2–11; part 2 of the UWDRS), with respect to activities of daily living, and neurological impairment (UWDRS_NI, items 12–35; part 3 of the UWDRS). On both subscales, higher scores indicated greater impairments.

### Copper metabolism

Serum concentrations of copper were measured by atomic absorption spectrophotometry, and serum ceruloplasmin concentrations were measured in an enzyme-based colorimetric assay [18]. The concentration of NCC was calculated according to a standard formula: NCC (μg/dl) = total serum copper (μg/dl) – 3.15 × serum ceruloplasmin (mg/dl) [1].

### Image acquisition and image analysis

All images were acquired according to a consistent protocol with a 1.5-T magnetic resonance imaging scanner (Achieva, Phillips, 8-channel head coil; see Suppl. Table 1 for sequence parameters). Based on the T1-weighted axial images, we estimated normalized brain volume (NBV), white matter (WM), gray matter (GM), peripheral gray matter (PGM), and ventricular cerebrospinal fluid (vCSF) with the cross*-*sectional version of Structural Image Evaluation using Normalization of Atrophy (SIENAX) provided in the Functional Magnetic Resonance Imaging of the Brain (FMRIB) software library [19]. Because some scans did not provide complete brain coverage, all SIENAX analyses were performed within a predefined range of standard space-Z coordinates (MNI space-Z coordinates between − 60 mm and 60 mm; ~ 95% of brain volume coverage, Suppl. Fig. 1). As MRI scans were not acquired prospectively with a consistent positioning protocol, in each case, we manually specified the center of gravity and used eye removal in the brain extraction step of SIENAX (options -c and -S). For all analyses, we used volumes that were normalized for head size by multiplying non-normalized volumes by subject-specific skull-scaling factors, derived with SIENAX [19, 20]. We calculated the brain parenchymal fraction (BPF) according to the following formula: BPF = NBV/(NBV + vCSF volume). The volumes of the thalami, caudate nuclei, globi pallidi, and putamina were estimated with FMRIB’s Integrated Registration and Segmentation Tool (FIRST) (Suppl. Fig. 1) according to a protocol optimized for two-dimensional images [21]. The total volume of these structures was used to estimate the volume of deep gray matter (DGM). In all cases, the results of brain image extraction and segmentation were visually inspected for quality control.

### Statistical analysis

Correlations between clinical measures, brain volumes, and laboratory measures were calculated with the Spearman rank correlation coefficient, *rho*; correlations were considered weak for *rho* ≤ 0.3, moderate for 0.3 < *rho* < 0.6, and strong for *rho* ≥ 0.6. To control for the effects of sex and age at diagnosis, we calculated partial Spearman correlation coefficients (*rho*_*p*_) and performed an analysis of covariance (ANCOVA). ANCOVAs were used to compare brain measures between sexes. The significance level was set at *p* < 0.05 (two-tailed). All analyses were performed with R (v3.3.2) software.

## Results

### Characteristics of participants

Demographic, clinical, and laboratory characteristics of participants are presented in Table 1. The mean age was 33.4 years and 54% were males. Approximately half (48%) of the patients studied had a predominantly neurological WD phenotype, with 6 patients exhibiting no symptoms. As expected, median serum ceruloplasmin concentrations were below the normal range, whereas mean NCC concentrations were above the upper limit of normal (15 μg/dL).

**Table 1:** Demographic, clinical, and laboratory characteristics of 48 patients with Wilson’s disease

Characteristic	Mean ± SD, unless otherwise stated
Mean age, years	33.37 ± 12.28
Gender, male, *N* (%)	26 (54)
Mean disease duration, years	
Predominantly hepatic	3.00 ± 2.47
Predominantly neurological	5.33 ± 6.98
Disease phenotype, *N* (%)	
Presymptomatic	6 (12)
Predominantly hepatic	19 (40)
Predominantly neurological	23 (48)
Predominant neurological form, *N* (% of patients with neurological symptoms)	
Ataxia/tremor	10 (43)
Parkinsonism	8 (35)
Dystonia	4 (17)
Unclassified	1 (4)
Kayser-Fleischer rings,	
Presymptomatic, *N* (%)	1(17)
Predominantly hepatic, *N* (%)	7 (58)
Predominantly neurological, *N* (%)	23(100)
Median UWDRS_NI score (range)	5 (0–46)
Median UWDRS_FI score (range)	0 (0–14)
Serum ceruloplasmin concentration, mg/dl; normal range: 25–45 mg/dl	13.6 ± 7.10^a^
Total serum copper concentration, μg/dl; normal range: 70–140 μg/dl	59.57 ± 21.27
Non-ceruloplasmin-bound copper concentration, μg/dl	16.05 ± 10.6^b^
Corneal copper deposits, *N* (%)	36 (75)

^a^We excluded data that were not acquired in our laboratory (one patient)

^b^We excluded data that were not acquired in our laboratory (one patient) and data of one patient with non-ceruloplasmin-bound copper concentration that was over 5 standard deviations above the mean (73.4 μg/dl)

### Relationship between brain volume parameters and age or sex

Age was significantly associated with decreased brain volume for NBV, BPF, GM, WM, PGM, and DGM but not for vCSF volume (Suppl. Table 2). Compared to women, men had a significantly lower age-adjusted volume of DGM (*p* = 0.002) and a significantly higher volume of vCSF (*p* = 0.044), and showed a trend of lower BPF values, although the difference was not statistically significant (*p* = 0.05). The remaining brain parameters did not differ significantly between sexes. None of the brain volumes correlated significantly with disease duration, also when corrected for sex and age (data not shown).

### Relationship between brain volume and functional and neurological impairments

**Fig. 1:**
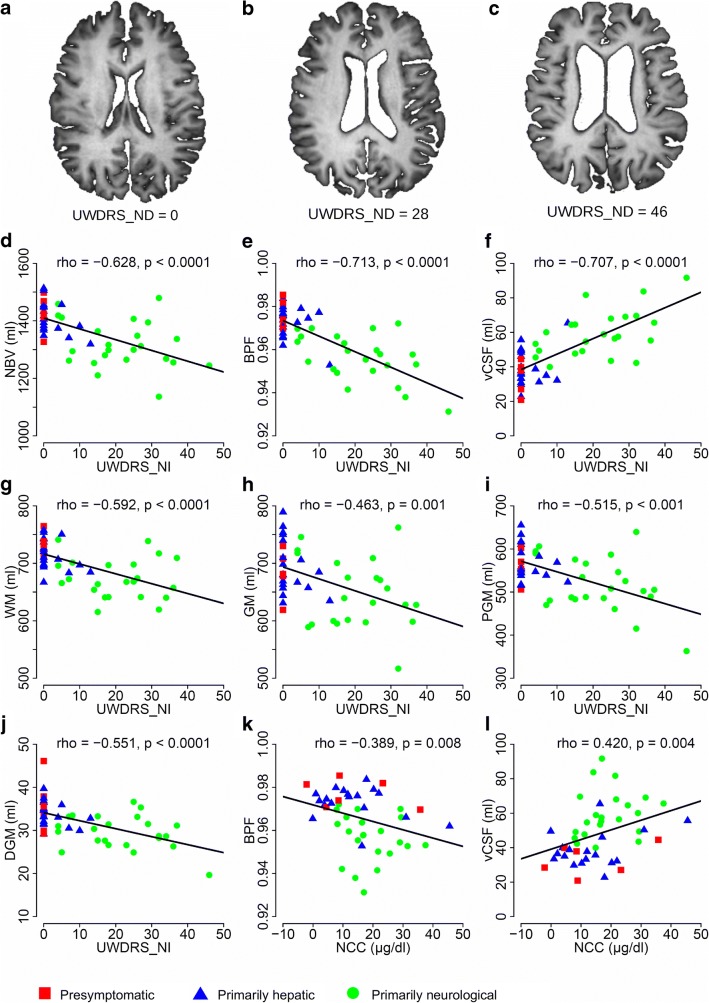
Relationships between brain volume measures, neurological impairment, and copper metabolism in patients with Wilson’s disease. **a**–**c** Representative brain magnetic resonance images of three patients with Wilson’s disease with different UWDRS_NI scores (UWDRS part 3). Images show that increasing brain atrophy is associated with increasing neurological impairment (higher UWDRS_NI scores). **d**–**j** Spearman rank correlation coefficients are shown for neurological impairment, based on UWDRS_NI scores, plotted against the NBV, BPF, and volumes of vCSF, WM, GM, PGM, and DGM. **k**, **l** Spearman rank correlation coefficients are shown for UWDRS_NI scores plotted against NCC, BPF, and the volume of vCSF. All correlation coefficients are non-parametric; regression lines indicate the direction of each relationship. UWDRS_NI Unified Wilson’s Disease Rating Scale—neurological impairment; NBV normalized brain volume; BPF brain parenchymal fraction; vCSF ventricular cerebrospinal fluid; GM gray matter; WM white matter; PGM peripheral gray matter; DGM deep gray matter; *r* Spearman’s rank correlation coefficient. In one patient, brain segmentation into total GM and WM volume was unsatisfactory, possibly due to severe brain atrophy; thus, this patient’s GM and WM data were excluded from the analysis

Neurological impairment, rated on the UWDRS_NI scale, correlated strongly with NBV, the BPF, and the vCSF volume (Fig. 1a–c). There were moderate correlations between the UWDRS_NI scores and the volumes of WM, GM, PGM, and DGM (Fig. 1g–j). Similarly, functional impairment, rated on the UWDRS_FI scale, correlated with all the analyzed brain volumes (Table 2). Importantly, nearly all correlations between the brain volume measures and scores of neurological and functional impairments remained significant after controlling for age and sex (*rho*_*p*_ values, Table 3).

**Table 2:** Spearman rank correlation coefficients for associations between functional impairment scores and brain volume measures in patients with Wilson’s disease

	NBV	BPF	vCSF	GM	WM	PGM	DGM
UWDRS_FI	− 0.388	− 0.622	0.634	− 0.236	− 0.399	− 0.325	− 0.458
	*p* = 0.006	*p* < 0.0001	*p* < 0.0001	*p* = 0.110	*p* = 0.005	*p* = 0.024	*p* = 0.001

*UWDRS_FI* Unified Wilson’s Disease Rating Scale—functional impairment, *NBV* normalized brain volume, *BPF* brain parenchymal fraction, *vCSF* ventricular cerebrospinal fluid, *GM* gray matter, *WM* white matter, *PGM* peripheral gray matter, *DGM* deep gray matter

**Table 3:** Partial Spearman rank correlation coefficients (*rho*_*p*_) for associations between functional and neurological impairment scores and brain volume measures (corrected for age and sex)

	NBV	BPF	vCSF	GM	WM	PGM	DGM
UWDRS_FI	− 0.383	− 0.609	0.623	− 0.236	− 0.390	− 0.297	− 0.442
	*p* = 0.009	*p* < 0.0001	*p* < 0.0001	*p* = 0.118	*p* = 0.008	*p* = 0.045	*p* = 0.002
UWDRS_NI	− 0.559	− 0.674	0.677	− 0.359	− 0.540	− 0.392	− 0.468
	*p* < 0.0001	*p* < 0.0001	*p* < 0.0001	*p* = 0.015	*p* < 0.001	*p* = 0.007	*p* = 0.001

*UWDRS_NI* Unified Wilson’s Disease Rating Scale—neurological impairment, *UWDRS_FI* Unified Wilson’s Disease Rating Scale—functional impairment, *NBV* normalized brain volume, *BPF* brain parenchymal fraction, *vCSF* ventricular cerebrospinal fluid, *GM* gray matter, *WM* white matter, *PGM* peripheral gray matter, *DGM* deep gray matter

### Relationship between copper metabolism, functional and neurological impairments, and brain volume

The NCC concentration was negatively correlated with both UWDRS_FI and UWDRS_NI scores (*rho* = − 0.295, *p* = 0.046 and *rho* = − 0.315, *p* = 0.033, respectively; age- and sex-adjusted *rho*_*p*_ = − 0.318, *p* = 0.035 and *rho*_*p*_ = − 0.382, *p* = 0.011, respectively). We found that the NCC concentration was significantly associated with the BPF (*rho* = − 0.389, *p* = 0.008) and the vCSF volume (*rho* = 0.420, *p* = 0.004; Fig. 1k and l). These correlations remained significant after controlling for age and sex (*rho*_*p*_ = − 0.456, *p* = 0.002 for BPF; *rho*_*p*_ = 0.475, *p* = 0.001 for vCSF). In addition, after controlling for age and sex, trends indicated that NCC could be associated with PGM (*rho*_*p*_ = − 0.295, *p* = 0.052). The remaining brain volume measures were not significantly associated with NCC (data not shown). We did not find any significant relationship between the analyzed brain volume measures and serum concentrations of ceruloplasmin or total copper.

## Discussion

This study for the first time investigated the relationship between brain volumes and disease severity and copper metabolism in WD. Our findings showed that the volumes of all the major brain structures correlated with functional and neurological impairments in treatment-naïve patients with WD. In addition, brain volume could be viewed as a marker of copper-induced neurodegeneration due to in WD, because lower brain volume was observed in patients with higher NCC concentrations.

Previous studies have attempted to investigate the relationship between neuroimaging results and the degree of neurological impairment in WD. However, they did not use validated tools, and/or they included heterogeneous patient samples (e.g., patients treated with different agents) [7, 22, 23]. In particular, previous studies did not investigate whether the severity of brain atrophy was related to the functional and neurological states of patients with WD or to NCC. Moreover, except for the study by Stezin [8], previous studies assessed brain atrophy in WD subjectively (presence vs. absence) [6, 7, 23]. We addressed these issues by evaluating neurological and functional states with a disease-specific clinical scale (UWDRS) and by quantitatively measuring brain volumes (with SIENAX and FIRST tools) in treatment-naïve patients with WD.

In our study, the total brain volume and the WM and GM volumes (both PGM and DGM; Fig. 1d–j) showed comparable relevance to the functional and neurological states of patients. This finding was consistent with the diffuse nature of brain atrophy described in patients with WD [5, 6, 9]. A likely explanation might be that copper accumulates equally in all brain regions in patients with WD [24], which leads to damage in both WM and GM; indeed, neuronal loss, axonal disruption, and multifocal demyelination are potential causes of brain atrophy [25, 26]. Moreover, in an earlier study, we found that greater neurological impairment severity in WD was associated with decreased retinal nerve fiber layer thickness [27]. That finding led us to suspect that, in WD, brain volume might be correlated with retinal nerve fiber layer thickness, as it is, for instance, in multiple sclerosis [28]. The strong correlation we found between brain volume and neurological and functional states suggested that brain volume measures could potentially become valuable endpoints in clinical trials in WD. This approach could decrease the sample size needed to demonstrate a treatment effect. However, measuring the effect would require longitudinal analyses to show that changes in brain volume over time were correlated with changes in the clinical state.

Similar to earlier studies, we found that age was associated with decreasing volumes of all the studied brain compartments [29, 30]. Also, in our study, male sex was a risk factor for decreased DGM and increased vCSF volumes. This added to our previous findings in WD that males, more frequently than females, showed neuroimaging abnormalities [6] and had a higher risk of developing neurological symptoms [31]. However, in this study, we could not find a feasible explanation for the inter-sex differences regarding the two brain volumes. First, these differences were not found in healthy individuals in a previous study that employed a similar method [32]. Second, deep brain nuclei were shown to be affected in WD to the same extent in both sexes [6]. Thus, further research is needed to evaluate deep brain nuclei in WD.

Copper toxicity is the key pathogenic mechanism in WD, and we showed herein that it was also related to brain atrophy and neurological and functional impairments at diagnosis. Indicators of total brain volume (i.e., BPF) and vCSF volume were significantly negatively and positively correlated, respectively, with the NCC concentration, which is typically elevated in untreated patients with WD. Similarly, PGM volume tended to be lower in patients with higher NCC concentrations.

Our study had limitations. First, it was retrospective, which in principle, can lead to selection bias, data inhomogeneity, and missing data. However, we collected neuroimaging and clinical data in our cohort in a prospective manner according to a standardized protocol. Therefore, we could retrieve data relevant to this study for all consecutive patients examined during the studied period. To preserve the homogeneity of data, we did not include copper metabolism data from patients treated with anti-copper agents or any data that were not acquired in our center. Second, in statistical terms, the study sample was small; however, WD is rare, and our sample of treatment-naïve patients was relatively large in comparison to earlier studies on WD. Third, the measurement of NCC concentration—one of the serum copper overload indices—was not standardized, which could lead to different findings between different laboratories [33]. However, all measurements of ceruloplasmin and copper in our study were consistently conducted in the same laboratory. Lastly, we did not assess cognitive function, which could be related to brain volume in WD.

In conclusion, our findings provided the first in vivo evidence that the severity of brain atrophy is an important correlate of neurological and functional impairments in patients with WD. Our findings also suggested that brain volume can serve as a marker of copper toxicity. In the future, longitudinal studies should be conducted to determine whether brain volume changes over time correlate with the changes in the clinical state.

## Electronic supplementary material

ESM 1 (DOCX 103 kb)

## References

[1] European Association for Study of Liver (2012). EASL clinical practice guidelines: Wilson’s disease. J Hepatol.

[2] Bandmann O, Weiss KH, Kaler SG (2015). Wilson’s disease and other neurological copper disorders. Lancet Neurol.

[3] Costa M do DL, Spitz M, Bacheschi LA (2009). Wilson’s disease: two treatment modalities. Correlations to pretreatment and posttreatment brain MRI. Neuroradiology.

[4] Saatci I, Topcu M, Baltaoglu FF, Köse G, Yalaz K, Renda Y, Besim A (1997). Cranial MR findings in Wilson’s disease. Acta Radiol.

[5] Sinha S, Taly AB, Prashanth LK, Ravishankar S, Arunodaya GR, Vasudev MK (2007). Sequential MRI changes in Wilson’s disease with de-coppering therapy: a study of 50 patients. Br J Radiol.

[6] Litwin T, Gromadzka G, Członkowska A, Gołębiowski M, Poniatowska R (2013). The effect of gender on brain MRI pathology in Wilson’s disease. Metab Brain Dis.

[7] Starosta-Rubinstein S, Young AB, Kluin K, Hill G, Aisen AM, Gabrielsen T, Brewer GJ (1987). Clinical assessment of 31 patients with Wilson’s disease. Correlations with structural changes on magnetic resonance imaging. Arch Neurol.

[8] Stezin A, George L, Jhunjhunwala K, Lenka A, Saini J, Netravathi M, Yadav R, Pal PK (2016). Exploring cortical atrophy and its clinical and biochemical correlates in Wilson’s disease using voxel based morphometry. Parkinsonism Relat Disord.

[9] Nakamura Y, Gaetano L, Matsushita T, Anna A, Sprenger T, Radue EW, Wuerfel J, Bauer L, Amann M, Shinoda K, Isobe N, Yamasaki R, Saida T, Kappos L, Kira JI (2018). A comparison of brain magnetic resonance imaging lesions in multiple sclerosis by race with reference to disability progression. J Neuroinflammation.

[10] Pini L, Pievani M, Bocchetta M, Altomare D, Bosco P, Cavedo E, Galluzzi S, Marizzoni M, Frisoni GB (2016). Brain atrophy in Alzheimer’s disease and aging. Ageing Res Rev.

[11] Guevara C, Bulatova K, Barker GJ, Gonzalez G, Crossley NA, Kempton MJ (2016). Whole-brain atrophy differences between progressive supranuclear palsy and idiopathic Parkinson’s disease. Front Aging Neurosci.

[12] Członkowska A, Tarnacka B, Möller JC et al (2007) Unified Wilson’s disease rating scale - a proposal for the neurological scoring of Wilson’s disease patients. Neurol Neurochir Pol 41:1–12 17330175

[13] Leinweber B, Möller JC, Scherag A, Reuner U, Günther P, Lang CJG, Schmidt HHJ, Schrader C, Bandmann O, Czlonkowska A, Oertel WH, Hefter H (2008) Evaluation of the unified Wilson’s disease rating scale (UWDRS) in German patients with treated Wilson’s disease. Mov Disord 23:54–62. 10.1002/mds.21761 10.1002/mds.21761 17960799

[14] Ferenci P, Caca K, Loudianos G, Mieli-Vergani G, Tanner S, Sternlieb I, Schilsky M, Cox D, Berr F (2003). Diagnosis and phenotypic classification of Wilson disease. Liver Int.

[15] Marsden CD (1987). Wilson’s disease. Q J Med.

[16] Oder W, Prayer L, Grimm G, Spatt J, Ferenci P, Kollegger H, Schneider B, Gangl A, Deecke L (1993). Wilson’s disease: evidence of subgroups derived from clinical findings and brain lesions. Neurology.

[17] Członkowska A, Litwin T, Dzieżyc K, Karliński M, Bring J, Bjartmar C (2018). Characteristics of a newly diagnosed polish cohort of patients with neurological manifestations of Wilson disease evaluated with the unified Wilson’s disease rating scale. BMC Neurol.

[18] Ravin HA (1961). An improved colorimetric enzymatic assay of ceruloplasmin. J Lab Clin Med.

[19] Zhang Y, Brady M, Smith S (2001). Segmentation of brain MR images through a hidden Markov random field model and the expectation-maximization algorithm. IEEE Trans Med Imaging.

[20] Smith SM, Jenkinson M, Woolrich MW, Beckmann CF, Behrens TEJ, Johansen-Berg H, Bannister PR, de Luca M, Drobnjak I, Flitney DE, Niazy RK, Saunders J, Vickers J, Zhang Y, de Stefano N, Brady JM, Matthews PM (2004). Advances in functional and structural MR image analysis and implementation as FSL. Neuroimage.

[21] Amann M, Andělová M, Pfister A, Mueller-Lenke N, Traud S, Reinhardt J, Magon S, Bendfeldt K, Kappos L, Radue EW, Stippich C, Sprenger T (2015). Subcortical brain segmentation of two dimensional T1-weighted data sets with FMRIB’s integrated registration and segmentation tool (FIRST). NeuroImage Clin.

[22] Sinha S, Taly AB, Ravishankar S, Prashanth LK, Venugopal KS, Arunodaya GR, Vasudev MK, Swamy HS (2006). Wilson’s disease: cranial MRI observations and clinical correlation. Neuroradiology.

[23] King AD, Walshe JM, Kendall BE, Chinn RJ, Paley MN, Wilkinson ID, Halligan S, Hall-Craggs MA (1996). Cranial MR imaging in Wilson’s disease. Am J Roentgenol.

[24] Litwin T, Gromadzka G, Szpak GM, Jabłonka-Salach K, Bulska E, Członkowska A (2013). Brain metal accumulation in Wilson’s disease. J Neurol Sci.

[25] Meenakshi-Sundaram S, Mahadevan A, Taly AB, Arunodaya GR, Swamy HS, Shankar SK (2008). Wilson’s disease: a clinico-neuropathological autopsy study. J Clin Neurosci.

[26] Mikol J, Vital C, Wassef M, Chappuis P, Poupon J, Lecharpentier M, Woimant F (2005). Extensive cortico-subcortical lesions in Wilson?s disease: clinico-pathological study of two cases. Acta Neuropathol.

[27] Langwińska-Wośko E, Litwin T, Dzieżyc K, Karlinski M, Członkowska A (2017). Optical coherence tomography as a marker of neurodegeneration in patients with Wilson’s disease. Acta Neurol Belg.

[28] Saidha S, Al-Louzi O, Ratchford JN (2015). Optical coherence tomography reflects brain atrophy in multiple sclerosis: a four-year study. Ann Neurol.

[29] Lee H, Prohovnik I (2008). Cross-validation of brain segmentation by SPM5 and SIENAX. Psychiatry Res.

[30] Goodro M, Sameti M, Patenaude B, Fein G (2012). Age effect on subcortical structures in healthy adults. Psychiatry Res.

[31] Litwin T, Gromadzka G, Członkowska A (2012). Gender differences in Wilson’s disease. J Neurol Sci.

[32] Király A, Szabó N, Tóth E, Csete G, Faragó P, Kocsis K, Must A, Vécsei L, Kincses ZT (2016). Male brain ages faster: the age and gender dependence of subcortical volumes. Brain Imaging Behav.

[33] Twomey PJ, Viljoen A, Reynolds TM, Wierzbicki AS (2008). Non-ceruloplasmin-bound copper in routine clinical practice in different laboratories. J Trace Elem Med Biol.

