# Supplementary material for: Brain volume is related to neurological impairment and to copper overload in Wilson’s disease
Source: Neurol Sci. 2019 May 30;40(10):2089–95. doi: 10.1007/s10072-019-03942-z (PMC6745045; doi:10.1007/s10072-019-03942-z)
Supplement: Supplementary file 1 — (DOCX 103 kb) [file 10072_2019_3942_MOESM1_ESM.docx]

**Supplemental data**

**1. METHODS**

**1.1 Image acquisition**

**Table 1**. Characteristics of magnetic resonance T1 sequences acquired in patients with Wilson’s disease

| Sequence type | 2D Spin Echo Pulse Sequence |
| --- | --- |
| Echo time (TE) [ms] | 15 |
| Repetition time (TR) [ms] | 596 |
| Flip angle [°] | 69 |
| Acquisition matrix [mm] | 256 × 205 |
| Field of view [mm] | 230 × 183 × 131 |
| Voxel size [mm] | 0.898 × 0.898 × 6 |

**1.2 Image analysis**

**1.2.1 SIENAX**

Based on the T1-weighted MRI scans, we estimated volumes of the brain, white matter, grey matter, peripheral grey matter, and ventricular cerebrospinal fluid with the cross*-*sectional version of the tool known as the Structural Image Evaluation using Normalization of Atrophy (SIENAX),[1] provided in the FMRIB software library.[2] Because some scans did not provide complete brain coverage, all SIENAX analyses were performed within a predefined range of standard space-Z coordinates (MNI space-Z coordinates between ‑60 mm and 60 mm; ~95% of brain volume coverage, Fig. e-1A, B). Because MRI scans were not acquired prospectively with a consistent positioning protocol, in each case, we manually specified the center of gravity and used eye removal in the brain extraction step of SIENAX (options -c and –S). In all cases, the results of brain extraction and segmentation were visually inspected for quality control. For all analyses, we used volumes that were normalized for head size by multiplying non-normalized volumes by subject-specific skull-scaling factors, derived with SIENAX.

**1.2.3 FIRST**

Volumes of selected subcortical structures (thalami, caudate nuclei, globi pallidi, and putamina; Fig. e1C) were estimated with FIRST (FMRIB's Integrated Registration and Segmentation Tool).[3] Before running FIRST, all images were interpolated to an isotropic resolution of 1 × 1 × 1 mm^3^ with FLIRT (FMRIB's Linear Image Registration Tool, also part of FSL)[4], as described by Amann et al.[5] Subsequently, the segmentation results produced by FIRST were corrected by subtracting ventricular system masks (derived with SIENAX) (Fig. 1C).[5] The obtained registration and segmentation results were inspected visually for quality control. Volumes were normalized for head size with the skull-scaling factor derived with SIENAX.


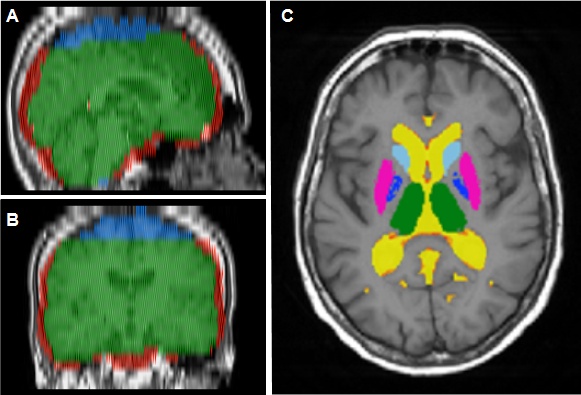


**Fig. 1. (**A-B) Brain extraction step with SIENAX; blue – brain mask; red – standard space mask within the range of predefined Z coordinates; green – combination of the brain mask and standard space mask. (C) Segmentation of deep grey matter nuclei performed with FIRST; green – thalami; magenta – putamina; light blue – caudate nuclei; dark blue – globi pallidi; yellow – cerebrospinal fluid.

**2. RESULTS**

**Table 2.** Spearman rank correlation coefficients for associations between age and brain volumes

|  | NBV | BPF | vCSF | GM | WM | PGM | DGM |
| --- | --- | --- | --- | --- | --- | --- | --- |
| Age | -0.680,  p<0.0001 | -0.324,  p=0.025 | 0.279,  p=0.055 | -0.699,  p<0.0001 | -0.348,  p=0.017 | -0.689,  p<0.0001 | -0.453,  p=0.001 |

NBV – normalized brain volume, BPF – brain parenchymal fraction, vCSF – ventricular cerebrospinal fluid, GM – grey matter, WM – white matter, PGM – peripheral grey matter, DGM – deep grey matter

**3. REFERENCES**

[1] S.M. Smith, Y. Zhang, M. Jenkinson, J. Chen, P.M. Matthews, A. Federico, N. De Stefano, Accurate, robust, and automated longitudinal and cross-sectional brain change analysis., Neuroimage. 17 (2002) 479–89. http://www.ncbi.nlm.nih.gov/pubmed/12482100 (accessed February 12, 2017).

[2] S.M. Smith, M. Jenkinson, M.W. Woolrich, C.F. Beckmann, T.E.J. Behrens, H. Johansen-Berg, P.R. Bannister, M. De Luca, I. Drobnjak, D.E. Flitney, R.K. Niazy, J. Saunders, J. Vickers, Y. Zhang, N. De Stefano, J.M. Brady, P.M. Matthews, Advances in functional and structural MR image analysis and implementation as FSL, Neuroimage. 23 (2004) S208–S219. doi:10.1016/j.neuroimage.2004.07.051.

[3] B. Patenaude, S.M. Smith, D.N. Kennedy, M. Jenkinson, A Bayesian model of shape and appearance for subcortical brain segmentation, Neuroimage. 56 (2011) 907–922. doi:10.1016/j.neuroimage.2011.02.046.

[4] M. Jenkinson, P. Bannister, M. Brady, S. Smith, Improved optimization for the robust and accurate linear registration and motion correction of brain images., Neuroimage. 17 (2002) 825–41. http://www.ncbi.nlm.nih.gov/pubmed/12377157 (accessed February 12, 2017).

[5] M. Amann, M. Andělová, A. Pfister, N. Mueller-Lenke, S. Traud, J. Reinhardt, S. Magon, K. Bendfeldt, L. Kappos, E.-W. Radue, C. Stippich, T. Sprenger, Subcortical brain segmentation of two dimensional T1-weighted data sets with FMRIB’s Integrated Registration and Segmentation Tool (FIRST), NeuroImage Clin. 7 (2015) 43–52. doi:10.1016/j.nicl.2014.11.010.
